# The isothiocyanate class of bioactive nutrients covalently inhibit the MEKK1 protein kinase

**DOI:** 10.1186/1471-2407-7-183

**Published:** 2007-09-25

**Authors:** Janet V Cross, Frank W Foss, Joshua M Rady, Timothy L Macdonald, Dennis J Templeton

**Affiliations:** 1 Department of Pathology, University of Virginia, Charlottesville, VA 22908, USA; 2 Department of Chemistry, University of Virginia, Charlottesville, VA 22908, USA

## Abstract

**Background:**

Dietary isothiocyanates (ITCs) are electrophilic compounds that have diverse biological activities including induction of apoptosis and effects on cell cycle. They protect against experimental carcinogenesis in animals, an activity believed to result from the transcriptional induction of "Phase 2" enzymes. The molecular mechanism of action of ITCs is unknown. Since ITCs are electrophiles capable of reacting with sulfhydryl groups on amino acids, we hypothesized that ITCs induce their biological effects through covalent modification of proteins, leading to changes in cell regulatory events. We previously demonstrated that stress-signaling kinase pathways are inhibited by other electrophilic compounds such as menadione. We therefore tested the effects of nutritional ITCs on MEKK1, an upstream regulator of the SAPK/JNK signal transduction pathway.

**Methods:**

The activity of MEKK1 expressed in cells was monitored using in vitro kinase assays to measure changes in catalytic activity. The activity of endogenous MEKK1, immunopurified from ITC treated and untreated LnCAP cells was also measured by in vitro kinase assay. A novel labeling and affinity reagent for detection of protein modification by ITCs was synthesized and used in competition assays to monitor direct modification of MEKK1 by ITC. Finally, immunoblots with phospho-specific antibodies were used to measure the activity of MAPK protein kinases.

**Results:**

ITCs inhibited the MEKK1 protein kinase in a manner dependent on a specific cysteine residue in the ATP binding pocket. Inhibition of MEKK1 catalytic activity was due to direct, covalent and irreversible modification of the MEKK1 protein itself. In addition, ITCs inhibited the catalytic activity of endogenous MEKK1. This correlated with inhibition of the downstream target of MEKK1 activity, i.e. the SAPK/JNK kinase. This inhibition was specific to SAPK, as parallel MAPK pathways were unaffected.

**Conclusion:**

These results demonstrate that MEKK1 is directly modified and inhibited by ITCs, and that this correlates with inhibition of downstream activation of SAPK. These results support the conclusion that ITCs may carry out many of their actions by directly targeting important cell regulatory proteins.

## Background

The MEKK1 protein kinase is a critical upstream mediator in signaling pathways that control the response of cells to stress stimuli. It directly phosphorylates and activates the SEK1 protein kinase, leading to activation of the stress activated protein kinase/jun N terminal kinase (SAPK/JNK) [1,2]. By virtue of its participation in this pathway, MEKK1 is involved in cellular responses to hyperosmotic shock, DNA damage and inflammatory cytokines [3,4]. It has also been characterized for its dual role in apoptosis signaling, contributing either a cell survival signal or a pro-apoptotic signal, depending on the form of the protein that predominates.

MEKK1 is a large protein kinase [5] with activity that is regulated by multiple diverse means including phosphorylation and proteolytic cleavage [6-9]. In addition, we recently demonstrated that MEKK1 is inhibited by oxidative stress stimuli through a mechanism involving direct glutathionylation of a specific cysteine residue in the ATP binding pocket [10]. This thiol modification is reversible by reducing agents, including glutathione, in vitro, and likely represents a reversible means of inhibiting the kinase activity within the cell during the response to oxidative insult.

The reactive cysteine in the ATP binding pocket of MEKK1 is quite unique among protein kinases. In an effort to identify cysteine reactive compounds that might likewise inhibit MEKK1 by targeting this residue, we considered physiologic agents that could result in protein modification on cysteine. One such group of compounds is the isothiocyanate (ITC) class of dietary chemopreventives, that have established roles in apoptosis and prevention of cancer, (for reviews, see [11-13]), processes in which MEKK1 has been implicated. These chemicals are abundant in members of the kale family, such as broccoli, and human studies have shown that consumption of broccoli sprouts  can result in circulating levels of ITCs in the low micromolar range [14].

Cancer chemoprevention by ITCs has been attributed to their ability to induce gene expression of a family of enzymes involved in detoxification and excretion of carcinogens, the Phase 2 genes [13]. However, this activity is insufficient to explain the ability of ITCs to induce growth arrest and apoptosis in tumor cells [15,16], to reduce tumorigenesis even if administered after the carcinogen [17,18], and to prevent tumor growth in xenograft models [19-21]. Instead, these results suggest that the ITCs may function by direct control of cell growth or death pathways.

The nature of the direct molecular target(s) of ITCs within cells has not been resolved. We reasoned that ITCs, being electrophilic compounds, could decorate cell regulatory proteins through stable covalent modification of cysteine or lysine residues, and that these targets could be identified using an affinity-labeled variant of nutritional ITCs. We tested this hypothesis on MEKK1, since the SAPK/JNK pathway that is regulated by MEKK1 has been implicated in apoptosis and cell growth, processes that are also impacted by ITCs.

We describe here results that indicate that nutritional isothiocyanates are able to covalently and irreversibly inhibit MEKK1. This inhibition is specific to MEKK1 and not to a closely related kinase, and occurs through covalent modification requiring a single cysteine in ATP binding pocket.

## Methods

### Chemicals and antibodies

Unless otherwise specified, all chemicals were obtained from Sigma-Aldrich. The Streptavidin-HRP detection reagent was from Zymed. *N,N*-dimethylformamide (DMF) and tetrahydrofuran (THF) were obtained from OptiDry canisters (<50 ppm H_2_O, Fisher Scientific). THF was passed through an activated alumina (activity I) column before use. DMF, MeCN (99.8% anhydrous; Aldrich; Milwaukee, WI), and chloroform were used without further drying. Norbiotinamine hydrochloride (Molecular Probes; Eugene, OR), EZ-Link™ 5-(biotinamido)-pentylamine (Pierce Biotechnology; Rockford, IL) were purchased and used as obtained. All other reagents, including 1,1'-thiocarbonyldi-2,2'-pyridone (TCDP), were purchased from Aldrich and used as obtained.

### Plasmids

MEKK1 and ASK1 plasmids and the C1238V mutant have been previously described [10].

### Cell Culture, Transfections and Treatments

LNCaP cells were grown in RPMI with 10% fetal bovine serum and penicillin/streptomycin antibiotics. For analysis of endogenous MEKK1, cells were serum starved for 18 hours prior to treatment. HeLa and CV-1 cells were grown in DMEM with 10% calf serum and penicillin/streptomycin antibiotics. For immunoblotting of active MAPKs, the cells were serum starved for 18 hours prior to treatment. Expression of proteins was accomplished using the T7-polymerase-driven vaccinia virus overexpression system [22] as previously described [10]. After overnight incubation, the cells were treated with isothiocyantes at the concentrations indicated in the figure legends for 20–30 minutes, then lysed for in vitro kinase assays or labeling reactions as described below.

### In vitro kinase assays

Following treatment, cells were lysed in MOPS lysis buffer (50 mM MOPS pH 7.0, 250 mM NaCl, 5 mM EDTA, 0.1% NP-40) supplemented with 2.5 μg/ml aprotinin, 2.5 μg/ml leupeptin, 10 mM sodium fluoride, 5 mM sodium pyrophosphate, 1 mM sodium orthovanadate, and 10 mM β glycerolphosphate. Lysates were clarified at 14,000 × g for 5 minutes in a microcentrifuge. CBD-tagged proteins were purified using chitin beads (New England Biolabs). The beads were washed 3 times with MLB, then once with 50 mM Tris/HCl (pH7.4). In some experiments, purified proteins were treated with isothiocyanates in vitro, in 50 mM Tris/HCl (pH7.4). Beads were then washed to remove isothiocyanates prior to kinase assay. Kinase assays were carried out in a buffer containing 50 mM Tris (pH7.4), 10 mM magnesium chloride, and 7.5 μM ATP in the presence of 10 μCi gamma^32^P-ATP. One μg of bacterially-expressed GST-tagged catalytically inactive SEK (GST SEK-KR) was used as substrate and the reactions were incubated for 30 minutes at room temperature. Kinase assays were stopped by addition of an equal volume of 2× Laemmli sample buffer (2% SDS, 100 mM Tris (pH 7.0), 20% Glycerol, 150 mM DTT, Bromophenol Blue), and then boiled and electrophoresed on SDS-PAGE (10%). Gels were transferred to PVDF (Immobilon) and imaged for quantification using a Packard Instant Imager. Equivalent protein was confirmed by immunoblotting with anti-MEKK1 (Santa Cruz Biotechnology).

### Synthesis of Bio-ITC [4-(4-Isothiocyanato-butyl)-tetrahydro-thieno[3,4-d]imidazol-2-one]

A solution of norbiotinamine hydrochloride (10 mg, 0.040 mmol) dissolved in acetonitrile (2 mL) and chloroform (0.40 mL) was prepared in a flame dried flask under Ar_(g)_. A solution of TCDP (10 mg, 0.040 mmol) in chloroform (0.40 mL) was added to the previously prepared solution. Following 15 minutes of continued stirring, one drop of triethylamine was added. The yellow-orange color was diminished upon addition. After 45 minutes, an additional drop of triethylamine was added and the reaction's progress was followed by thin layer chromatography (TLC). After four hours, the starting material was consumed and the reaction was concentrated to a crude material that was purified by normal phase preparative-TLC (Silica Gel GF^®^, 500 microns, Analtech) using 1:9 methanol/chloroform as an eluent. Following separation, the desired band was carefully isolated from the plate and washed through a fritted funnel with multiple portions of ethyl acetate. The organic solvent was concentrated to 7 mG (70%) of the title compound as a off-white powdery solid. R_f _(10% MeOH in CHCl_3_) = 0.22. ^1^H NMR (300 MHz, CD_3_OD, 23°C, δ): 4.50 (dd, *J *= 7.9, 5.1 Hz, 1 H), 4.33 (dd, *J *= 7.1, 4.5 Hz, 1 H), 3.58 (t, *J *= 6.5 Hz, 2 H), 2.95 (dd, *J *= 12.8, 5.1 Hz, 1 H), 2.72 (d, *J *= 12.8 Hz, 1 H), 1.83–1.52 (m, 6 H) ppm. ^13^C NMR (300 MHz, CD_3_OD, 23°C, δ): 168.30, 125.85, 63.41, 61.63, 56.90, 41.05, 31.00, 29.08, 27.33 ppm. MS (ESI+) *m/z *258 [M+H]^+^.

### BioITC labeling reactions

Untreated or PEITC treated cells were lysed in Tris lysis buffer (50 mM Tris pH7.8, 0.1% NP-40) supplemented with 2.5 μg/ml aprotinin and 2.5 μg/ml leupeptin. Lysates were clarified by centrifugation at 14,000 × g for 5 minutes. Clarified lysates were reacted with 500 μM Bio-ITC for 30 minutes at room temperature. Samples were removed for in vitro kinase assay, cell lysate immunoblots, and/or chitin bead purification and immunoblot analysis, as indicated in the figure legends.

### Analysis of MAPK pathways

HeLa cells were seeded in 6 well plates and then serum starved overnight. PEITC at concentrations of 200 μM to 12.5 μM (in two fold serial dilutions) was added to the media and the cells incubated for 7 minutes at 37°C. Sorbitol was then added to 400 mM to induce hyperosmotic stress, and the incubation continued for an additional 20 minutes. Cells were lysed in 500 μL MLB, and 200 μL used to prepare a whole cell lysate by adding 50 μL of 5× Laemmli sample buffer. Samples were analyzed by immunoblotting using antibodies directed against phosphorylated forms of various MAPK family members as described in the figure legend.

## Results

### ITCs inhibit MEKK1, but not the related protein kinase ASK1, in intact cells

**Figure 1:**
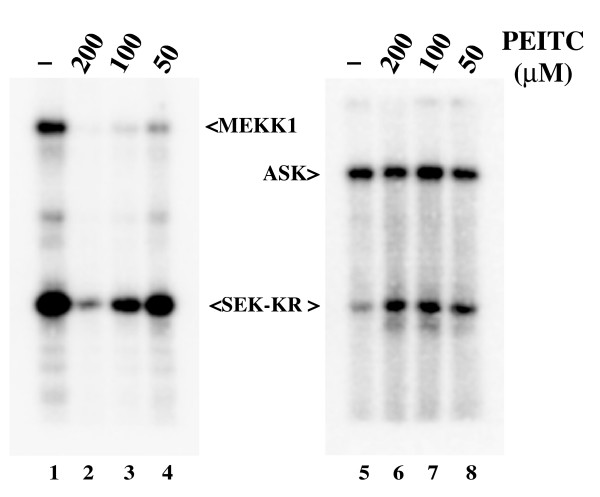
**PEITC Inhibits MEKK1, but not ASK1**. CV-1 cells expressing CBD-tagged full-length MEKK1 (left panel) or ASK1 (right panel) were either left untreated (lane 1 and 5) or treated with PEITC at the concentrations indicated (lanes 2–4 and 6–8 respectively) for 30 minutes at 37°C. CBD-tagged proteins were purified on chitin beads and assayed for kinase activity using SEK-KR as substrate. MEKK1 was dose responsively inhibited by PEITC. The related ASK1 kinase was completely unaffected by PEITC, indicating that the inhibition of MEKK1 is specific.

To determine the effect of isothiocyanates on MEKK1 activity, we expressed a full-length MEKK1 protein fused to a chitin binding domain (CBD) tag to allow purification of the protein by binding to chitin beads. In parallel, we expressed the related related MAPK3K, the Apoptosis Signal-regulating Kinase (ASK1), bearing the same carboxy-terminal CBD tag. The cells were treated with increasing concentrations of phenethyl isothiocyanate (PEITC), and each kinase was purified by affinity chromatography using chitin beads followed by *in vitro *kinase assay using a bacterially-expressed inactive mutant of SEK (SEK-KR) as substrate. Treatment of intact cells with PEITC inhibited MEKK1 in a dose dependent fashion within 30 minutes of exposure (Figure 1, left panel). In contrast, ASK1 was not inhibited by PEITC treatment, even at the highest concentrations (Figure 1, right panel), demonstrating a specific mechanism of inhibition of MEKK1 by PEITC. We observed inhibition of MEKK1 by other ITCs that have been implicated in cancer chemoprevention, including sulforaphane, at similar concentrations (not shown).

### Inhibition of MEKK1 by PEITC is direct and requires cysteine 1238

**Figure 2:**
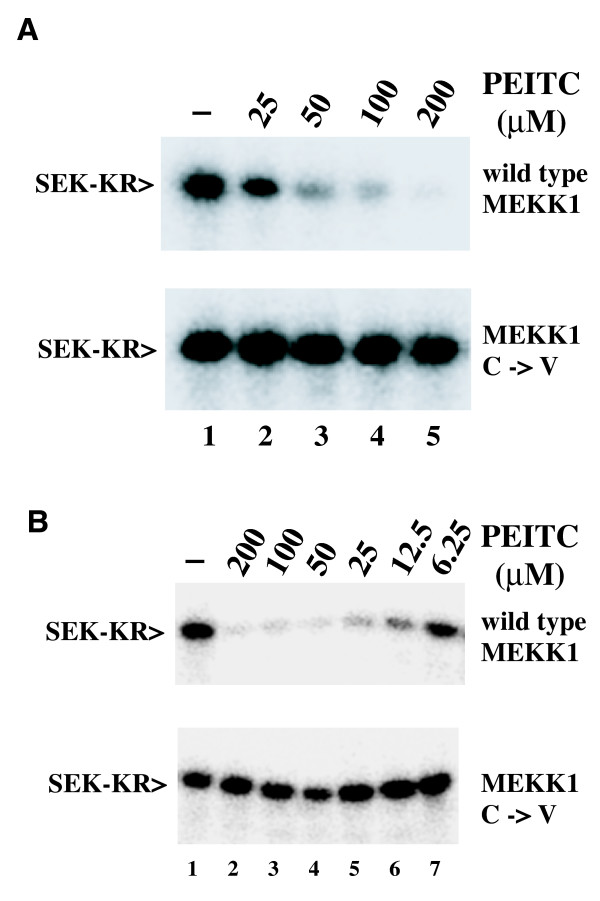
**PEITC inhibits wild type MEKK1, but not the C1238V mutant in cells and *in vitro***. **A**. CV-1 cells expressing the catalytic domain fragment of wild type MEKK1 (top panel) or the C1238V mutant (bottom panel) were treated with PEITC at the indicated concentrations for 1 hour at 37°C. Kinases were purified on chitin beads and assayed for activity by in vitro kinase assay. **B**. Purified wild type MEKK1 (top panel) or the C1238V mutant (bottom panel) were treated with PEITC at the indicated concentrations for 30 minutes at room temperature. PEITC inhibited wild type MEKK1, but not the C1238V mutant both after treatment of intact cells and treatment of purified protein *in vitro*, indicating that the inhibition of MEKK1 is a direct effect on the kinase itself and requires the cysteine at position 1238.

We previously identified a cysteine residue in the ATP binding pocket of MEKK1 that is glutathionylated during oxidative stress, resulting in inhibition of the kinase [10]. We compared the inhibition of the wild type catalytic domain fragment of MEKK1 to the effect of ITCs on a mutant MEKK1 protein in which C1238 has been mutated to valine. The C1238V mutant retains full wild type activity and is not inhibited by oxidative stress stimuli. PEITC dose dependently inhibited the wild type catalytic fragment (Figure 2A, top panel). However, the C1238V mutant was completely resistant to treatment with PEITC (Figure 2A, bottom panel). Thus, inhibition of MEKK1 by PEITC requires the cysteine residue at position 1238.

To determine whether inhibition of MEKK1 is due to direct modification of MEKK1 and not an effect on another member of the signaling pathway, we examined the sensitivity of purified MEKK1 to PEITC treatment *in vitro*. CBD-tagged wild type MEKK1 or the C1238V mutant were expressed in cells, purified on chitin beads and then treated with PEITC *in vitro*. As observed in experiments with intact cells, wild type MEKK1 was inhibited by PEITC treatment in a dose dependent manner, while the C1238V mutant was completely resistant to PEITC treatment (Figure 2B). This demonstrates that inhibition of MEKK1 by PEITC is due to a direct effect on the kinase itself, and not due to inhibition of another component of the signaling pathway.

### MEKK1 is protected from PEITC inhibition by an ATP analog

**Figure 3:**
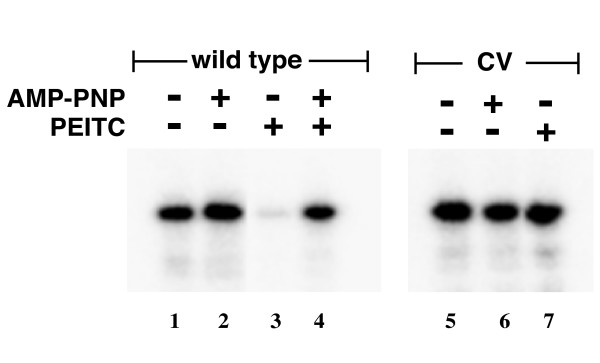
**AMP-PNP interferes with PEITC inhibition of MEKK1**. MEKK1 catalytic domain fragment or the C1238V mutant were purified on chitin beads and then preincubated with 2 mM AMP-PNP/10 mM MgCl2 in 50 mM Tris pH7.8 for 15 minutes at room temperature. PEITC was added to the reactions, and the incubations continued for an additional 30 minutes. The bead bound proteins were then washed to remove the agents and kinase activity measured. Preincubation with AMP-PNP protected MEKK1 from inhibition by subsequent exposure to PEITC in vitro. The C1238V mutant was unaffected by any of the treatments.

Since C1238 is within the ATP binding domain of MEKK1, we suspected that occupancy of the ATP-binding pocket of MEKK1 might render MEKK1 resistant to modification by PEITC. We found that pre-loading the MEKK1 with the non-hydrolyzable ATP analog AMP-PNP, *in vitro*, prior to PEITC exposure, protected MEKK1 from inhibition by PEITC (Figure 3, left panel). Again, the C1238V mutant was resistant to PEITC (Figure 3, right). Thus PEITC inhibition of MEKK1 requires both access to the ATP-binding pocket of MEKK1 and the cysteine at position 1238.

### MEKK1 is covalently labeled by a new ITC probe: Biotin-ITC

**Figure 4:**
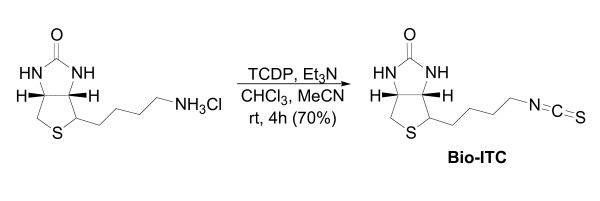
**Synthesis of Bio-ITC**. Norbiotinamine hydrochloride was converted to the isothiocyanate, **Bio-ITC**, by mild conditions with 1,1'-thiocarbonyldi-2,2'-pyridone (TCDP) in the presence of an amine base (Et_3_N). Synthesis and characterization are described in detail in the materials and methods.

Using a modification of a published procedure [23], we designed and synthesized an isothiocyanate molecule that contained a biotin moiety, termed Bio-ITC, to enable specific detection and potential affinity purification of targets of covalent ITC modification (Figure 4). The 1H NMR spectrum was consistent with published data. Bio-ITC was further characterized by 13C NMR and low-resolution mass spectrometry.

**Figure 5:**
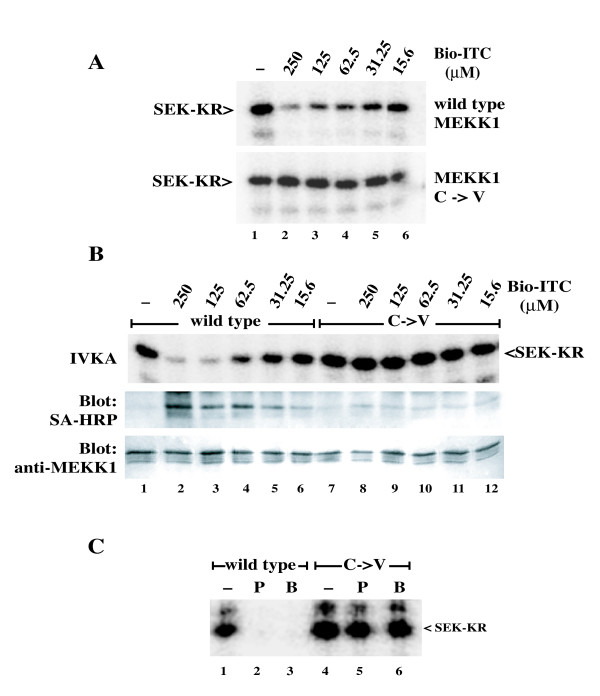
**BioITC inhibits MEKK1, but not the C1238V mutant, by covalent modification**. **A**. Purified MEKK1 or the C1238V mutant on chitin beads were treated with Bio-ITC at the concentrations indicated in 50 mM Tris pH7.8 for 40 minutes at room temperature. Bio-ITC was removed, and the kinase activity measured. Bio-ITC inhibits wild type MEKK1, but not the C1238V mutant in a dose dependent manner. **B**. Purified wild type MEKK1 or the C1238V mutant were treated as in Panel A with Bio-ITC at the indicated concentrations. The treated protein was used to either assay for kinase activity (top panel) or to detect covalent modification by Bio-ITC using a streptavidin-HRP conjugate (middle panel). Equivalent protein was confirmed by re-probing the streptavidin gel with anti-MEKK1 (bottom panel), using an alkaline phosphatase conjugated secondary antibody and colorimetric detection reagents. Bio-ITC inhibited wild type MEKK1 by stable, covalent modification in a manner that requires C1238V. **C**. CV-1 cells expressing either wild type MEKK1 or the C1238V mutant were lysed in Tris lysis buffer containing 0.1% NP-40. Clarified lysates were treated by addition of either PEITC (P) or Bio-ITC (B) to final concentration of 250 μM, and incubated at room temperature for 30 minutes. MEKK1 proteins were purified on chitin beads and kinase activity measured. Both PEITC and Bio-ITC completely inhibited wild type MEKK1, but not the C1238V mutant when added to cell lysates.

Figure 5 demonstrates that Bio-ITC is functionally similar to other isothiocyanates. Bio-ITC dose dependently inhibited the activity of purified MEKK1 *in vitro *(Figure 5A, top), without inhibiting the C1238V mutant (Figure 5A, bottom). Bio-ITC covalently labeled MEKK1, since dose dependent inhibition of MEKK1 (Figure 5B, top) resulted in wild type MEKK1 that was labeled with biotin, as demonstrated by detection with a streptavidin-enzyme conjugate (Figure 5B, middle). The C1238V mutant was neither inhibited by Bio-ITC nor reactive with the streptavidin conjugate. This demonstrates both that C1238 is required for modification of MEKK1 by Bio-ITC, and also that none of the other cysteines of MEKK1 can be stably modified by Bio-ITC.

In contrast to the natural isothiocyanates, the charged Bio-ITC was, as expected, poorly cell permeant. However, wild type MEKK1, but not the C1238V mutant, was inhibited by Bio-ITC in detergent-permeablized cell lysates (Figure 5C, lanes 3 and 6). The pattern of inhibition was similar to that observed with the natural isothiocyanate, PEITC (lanes 2 and 5).

**Figure 6:**
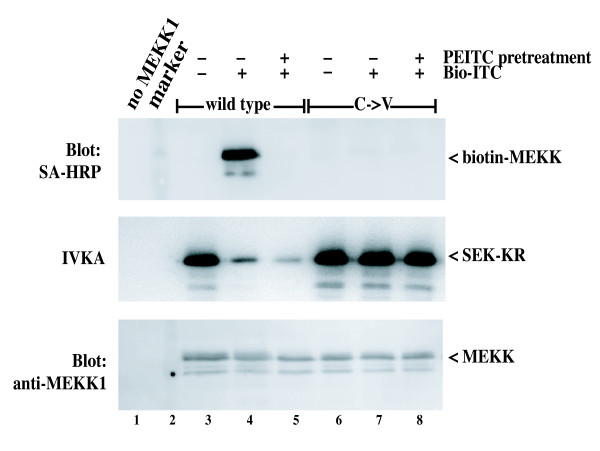
**Covalent modification of MEKK1 by Bio-ITC is blocked by preincubation of cells with PEITC**. CV-1 cells expressing wild type MEKK1 or the C1238V mutant were either left untreated or incubated with 250 μM PEITC for 20 minutes at 37°C. Lysates were prepared in TLB, clarified and then 500 μM Bio-ITC was added to the samples as indicated. MEKK1 proteins were purified from the lysates on chitin beads, and either electrophoresed for detection of biotin labeling using streptavidin-HRP (top panel) or assayed for kinase activity (middle panel). To confirm equivalent protein, the SA-HRP blot was re-probed with anti-MEKK1 as in Figure 5. Lane 2 contains the molecular weight markers. Bio-ITC covalently modifies MEKK1 protein and inhibits its activity in a manner that requires C1238V. The modification by Bio-ITC is completely inhibited by preincubation of the cells with PEITC, suggesting that the modification site is completely occupied by the natural isothiocyanate after exposure of intact cells.

We used a blocking strategy to demonstrate the specificity of *in vitro *labeling with Bio-ITC (Figure 6). Pretreatment of intact cells with PEITC prior to preparation of the lysates inhibited the kinase activity of the MEKK1 protein and prevented subsequent *in vitro *labeling of wild type MEKK1 by Bio-ITC (lane 5 middle and top panel, respectively). As before, wild type MEKK1 but not C1238V MEKK1 was inhibited *in vitro *by Bio-ITC (lanes 4 and 7, middle panel), and wild type MEKK1 (but not C1238V MEKK1) was covalently labeled with Bio-ITC (lanes 4 and 7, top panel). These results suggest that both Bio-ITC and PEITC inhibit MEKK1 by direct modification in a manner that requires the cysteine at position 1238.

### PEITC specifically inhibits endogenous MEKK1 and downstream stress signaling kinases

**Figure 7:**
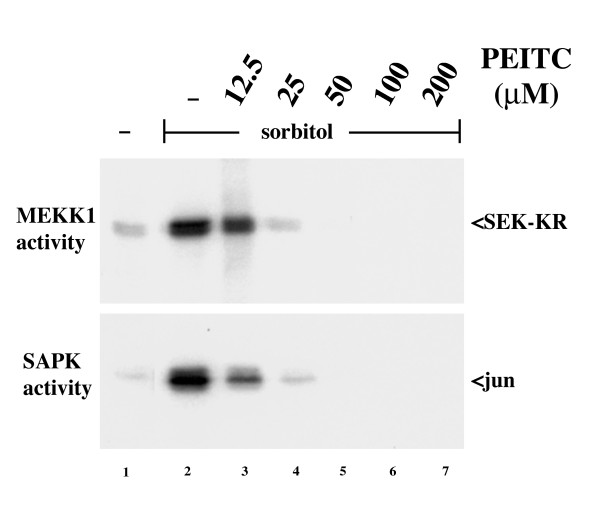
**PEITC inhibits endogenous MEKK1 and downstream signaling to SAPK**. LNCaP cells were incubated with PEITC at the indicated concentrations for 15 minutes at 37°C. Hyperosmotic shock was induced by addition of sorbitol to a final concentration of 300 mM and cells were incubated for an additional 15 min. Lysates were prepared, MEKK1 and SAPK were isolated by immunoprecipitation and their activity was measured by in vitro kinase assay with SEK-KR (MEKK1, upper panel) or jun (SAPK, lower panel) as substrate. Activation of endogenous MEKK1 by hyperosmotic shock was dose dependently inhibited by PEITC pretreatment. The inhibition of MEKK1 exactly paralleled inhibition of SAPK.

We examined the effect of PEITC pretreatment on activation of endogenous MEKK1 by hyperosmotic shock in LnCAP cells. These cells were chosen because they have relatively high expression of MEKK1 in comparison to other cell lines, allowing detection of the endogenous protein activity by immunoprecipitation and *in vitro *kinase assay. Hyperosmotic shock, induced by addition of 0.3 M sorbitol, strongly activated MEKK1 (Figure 7, upper panel). Pretreatment of cells with PEITC dose-dependently inhibited this activation, with complete inhibition at the 50 μM dose, nearly complete inhibition at 25 μM, and partial inhibition at 12.5 μM. This inhibition exactly paralleled the effect of PEITC on SAPK activity in the same lysates (Figure 7, lower panel). Thus PEITC blocks activation of MEKK1 by osmotic shock, and this inhibition exactly correlates with inhibition of SAPK.

**Figure 8:**
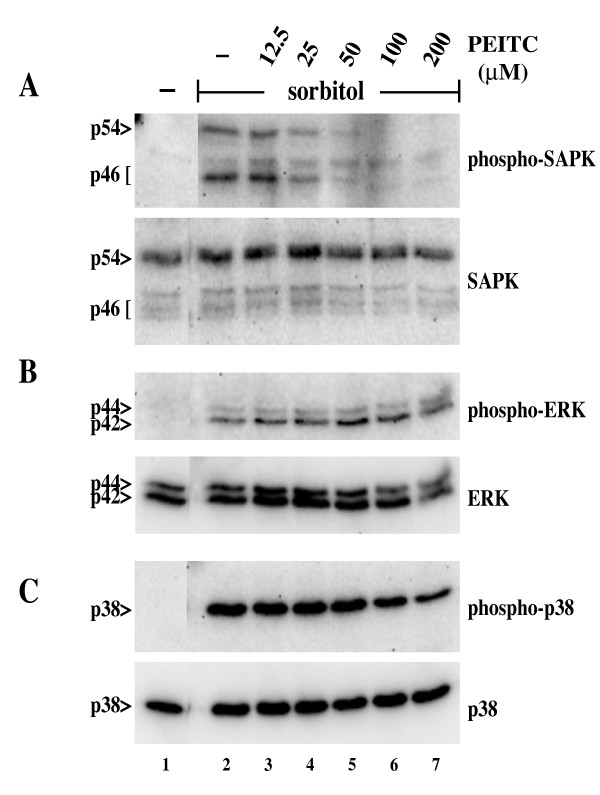
**PEITC specifically interferes with downstream signaling to SAPK/JNK**. HeLa cells were incubated with PEITC at the indicated concentrations for 7 minutes at 37°C. Hyperosmotic shock was induced by addition of sorbitol to a final concentration of 400 mM, and the cells incubated for an additional 20 minutes. Cell lysates were analyzed by immunoblotting with antibodies for phosphorylated forms of the MAPKs as indicated to the right. Duplicate gels were probed with antibodies to each MAPK, to confirm equivalent protein expression. PEITC interferes with the activation of SAPK, but not ERK or p38, in a dose dependent manner.

To test the specificity of PEITC in inhibiting the SAPK signaling pathway, we examined the impact of PEITC pretreatment on the activation of other MAPKs by hyperosmotic shock. As expected, we observed that PEITC specifically inhibited the activating phosphorylation of SAPK induced by hyperosmotic shock using sorbitol (Figure 8, Panel A), consistent with the inhibition seen by *in vitro *kinase assay. In contrast, moderate concentrations of PEITC had no effect on the sorbitol-induced phosphorylation and activation of the related MAPK family members, ERK1/2 and p38 (Figure 8, Panel B and C, respectively). We did observe fractional inhibition of p38 phosphorylation at the highest concentration of PEITC. However, p38 is substantially less sensitive to PEITC mediated inhibition than SAPK. Thus, inhibition of MEKK1 by PEITC specifically inhibits downstream stress signals leading to SAPK, but not to other MAPK pathways.

## Discussion

Our data show that both endogenous and plasmid-expressed wild type MEKK1 are dose-dependently inhibited by ITCs, through stable, direct covalent modification in a manner requiring C1238. Signaling downstream of endogenous MEKK1 is also specifically inhibited by PEITC, since the MEKK1 target kinase, SAPK/JNK is inhibited at similar doses, while ERK and p38 MAP kinases are not inhibited. This result supports the conclusion that SAPK activating pathways are specifically silenced by inhibition of MEKK1 or similarly regulated kinases.

While the MEKK1-related kinase ASK1 is associated with initiation of apoptotic pathways, MEKK1 itself is associated with anti-apoptotic responses [8,9]. This has been confusing, since MEKK1 and ASK1 are thought to activate similar if not identical downstream pathways. Specific inhibition of MEKK1, but not ASK1, by PEITC is compatible with the pro-apoptotic function of PEITC that might be enabled by this differential effect on protein kinase pathways.

Inhibition of endogenous MEKK1 activity in intact LnCAP cells requires a relatively high concentration of PEITC compared to the dietary plasma levels in humans following a single oral dose of ITCs (measured at about 2 μM [14]). However, partial inhibition of MEKK1 at 12.5 μM, with nearly complete inhibition at 25 μM and complete abrogation at 50 μM, is remarkably similar to the dose response reported in numerous studies examining apoptotic effects of PEITC and correlating these effects with a variety of markers of the apoptotic response [24-32].

Inhibition of MEKK1 activity within 30 minutes of PEITC addition indicates that inhibition is a specific and acute effect that is not due to protein loss that might accompany measurements taken at later time points after apoptosis had commenced. This is supported by equivalent recovery of MEKK1 protein from the treated and untreated samples. *In vitro *inhibition of MEKK1 by PEITC also supports a direct effect on catalytic activity, rather than a more complicated mechanism of regulation involving protein degradation or other regulators of MEKK1 function. Use of the Bio-ITC probe conclusively establishes the mechanism for this inhibition as covalent modification of C1238 (but not other cysteines) of MEKK1.

Covalent modification of cellular proteins by ITCs has been hypothesized previously. One group used indirect assays to conclude that the Keap1 protein transiently reacts with sulforaphane [33]. Since Keap1 is involved in regulation of the activity of the Nrf2 transcription factor that directs Phase 2 gene transcription from the promoter element termed the antioxidant response element (ARE), Keap1 is an attractive potential target for ITCs. However, in this study, while spectrophotometric assays of absorbance shifts were used to support the conclusion that Keap1 is modified by sulforaphane, no direct evidence was presented for covalent labeling of Keap1. In addition, these studies used purified recombinant Keap1 *in vitro*, and no evidence was presented to support the ability of sulforaphane to modify Keap1 in intact cells.

While the Keap1 study predicted that ITCs should make readily-reversible reaction products with protein sulfhydryls, our results with MEKK1 demonstrate stable products that are resistant to heat, denaturants, and SDS used in protein electrophoresis. While transient interaction of ITCs with some protein thiols (as well as with glutathione) likely occur in intact cells, stable covalent modifications of proteins are more likely to represent functional targets, particularly at low ITC concentration. Our identification of MEKK1 as a functional target of ITCs points the way to an understanding of a general mechanism of ITCs and other bioactive electrophiles. It remains unproven whether modification and inhibition of MEKK1 plays a singular role in the effects of ITCs in apoptosis or cancer chemoprevention. We suspect that MEKK1 is one of several or many cellular targets that contribute to the multiple effects of ITCs. Identification of these targets and characterization of their relative role(s) in the transcriptional, cell growth and apoptosis pathways induced by ITCs is an important goal for future studies. We are currently using our Bio-ITC reagent and affinity chromatography to identify other stably modified proteins that could, as a whole, explain the means through which these dietary constituents prevent cancer.

After the completion of this work, reports were published that ITCs and other thiol reactive electrophilic compounds are able to covalently modify and activate the TRPA1 ion channel involved in pain sensing [34,35]. The two groups disagreed about whether the modifications were reversible and which cysteines were required for the modification, and neither group considered the potential role of this protein target in chemoprevention. However, these data provide an additional model for ITCs specifically and covalently modifying protein targets on cysteine residues, supporting our contention that direct modification of protein targets by dietary ITCs may play an important role in their chemopreventive activity. We expect that several covalent targets of ITCs may together contribute to the multiple biological functions of these important nutritional components.

## Conclusion

Our results provide new insights into the molecular actions of dietary isothiocyanates. ITCs directly and covalently modify at least one cell signaling protein within cells, resulting in the functional silencing of the MEKK1 protein kinase and blockade of SAPK/JNK signaling. Our results do not preclude ITCs having other functional targets in cells that may contribute to the pleiotropic functions of these nutritional compounds. However, we predict that all cellular targets of ITCs will parallel the mechanism of MEKK1 inactivation, i.e. through stable covalent modification resulting in altered protein function.

## Abbreviations

ITC(s), isothiocyanate(s); MEKK1, MAPK/ERK kinase 1; SEK, SAPK/ERK kinase; SAPK/JNK, Stress Activated Protein Kinase/jun N-terminal kinase; CBD, chitin binding domain; MLB, MOPS lysis buffer; GST, glutathione S-transferase; Bio-ITC, Biotin-ITC; TLB, Tris lysis buffer; PEITC, phenethylisothiocyanate; ERK, extracellular signal regulating kinase; ARE, Antioxidant Response Element; ASK1, Apoptosis signal regulating kinase 1.

## Competing interests

The author(s) declare that they have no competing interests.

## Authors' contributions

JVC and DJT designed and interpreted the experiments. JVC performed most of the experiments. JMR assisted with design/execution of some experiments. FWF, Jr. and TLM perfected and executed the synthesis of Bio-ITC. JVC and DJT wrote the manuscript. All authors have read and approved the final version of the manuscript.

## Pre-publication history

The pre-publication history for this paper can be accessed here:


